# Combination of Osimertinib and Olaparib Therapy to Treat Non-Small Cell Lung Cancer and High-Grade Serous Ovarian Carcinoma: A Case Report

**DOI:** 10.3390/curroncol31010039

**Published:** 2024-01-19

**Authors:** Jane Lin, Stephen Welch, Michael Sanatani, Sherif Ramadan

**Affiliations:** 1 Schulich School of Medicine & Dentistry, Western University, London, ON N6A 3K7, Canada; jlin2025@meds.uwo.ca; 2 Division of Medical Oncology, Department of Oncology, Western University, London, ON N6A 3K7, Canada; michael.sanatani@lhsc.on.ca; 3 Postgraduate Medical Education, Department of Oncology, Western University, London, ON N6A 3K7, Canada; sherif.ramadan@lhsc.on.ca

**Keywords:** non-small cell lung cancer, osimertinib, ovarian serous carcinoma, olaparib, combination drug therapy

## Abstract

We present the case of a 75-year-old female with simultaneous EGFR-mutated stage IV lung cancer and advanced BRCA2-mutated ovarian cancer, treated with a unique regimen. In this case report, the patient was treated with alternating months of osimertinib and olaparib to control her lung and ovarian cancers, respectively. When both diseases showed progression, the patient underwent a trial of concurrent therapy with both drugs, yet this was discontinued due to patient-reported adverse side effects. Combination targeted drug therapy may be required to treat complex diagnoses such as dual malignancies. However, combination drug therapy consisting of osimertinib and olaparib has not previously been explored. This case report represents the first to demonstrate osimertinib and olaparib combination therapy as a unique treatment regimen for concurrent lung and ovarian cancers. These two drugs can either be given in an alternating way or given together, short-term, with a higher but tolerable toxicity profile.

## 1. Introduction

Patients with two synchronous primary malignancies are rare and complicated to treat. The incidence of multiple primary cancers varies between 2–17% [1]. In particular, patients with the dual diagnoses of lung and ovarian cancers are even more rare, and so far, no literature data are available on the incidence of this double primary. In advanced stages, prognoses are poor for both malignancies [2].

Lung cancer is the second most common cancer throughout the world, and non-small cell lung cancer (NSCLC) accounts for 84% of lung cancers [3]. Chemotherapy, radiation therapy, and surgery remain the mainstays of lung cancer treatment. The treatment decisions for NSCLC are also influenced by molecular driver mutations. Epidermal growth factor receptor (EGFR) mutations are the most common driver mutations. Gefitinib is a first-generation EGFR tyrosine kinase inhibitor (TKI), which reversibly binds to EGFR to block cell proliferation and results in cell death [4]. Afatinib, a second-generation EGFR-TKI, is an irreversible inhibitor that covalently binds to EGFR [4]. Both gefitinib and afatinib are approved as first-line treatments for EGFR-mutant tumors. The T790M mutation is a common cause of acquired resistance to EGFR-TKI. Osimertinib, a third-generation EGFR-TKI, can selectively target mutant EGFRs that include T790M mutations. NSCLC patients with T790M mutations and resistance to first-generation EGFR-TKI have shown significant improvement when started on osimertinib [5,6]. In addition, osimertinib can effectively penetrate the blood-brain barrier, which is beneficial, as central nervous system (CNS) metastases are commonly observed in NSCLC patients [7].

Ovarian cancer remains one of the most prevalent cancers in women and is the leading cause of death from gynecologic cancers. BRCA1/2 mutations are common driver mutations leading to serous carcinoma, which is the most common type of ovarian cancer [8]. Treatments for BRCA-mutated ovarian cancer include first-line platinum-based chemotherapy and cytoreductive surgery. In addition, olaparib is a poly ADP-ribose polymerase (PARP) inhibitor that prevents the DNA repair mechanisms in cells. Olaparib is approved as a first-line maintenance monotherapy and as maintenance therapy after the response to chemotherapy for platinum-sensitive relapse [9,10].

In this report, we describe a patient with metastatic EGFR-mutated NSCLC and metastatic BRCA-mutated ovarian serous carcinoma who has been treated with alternating osimertinib and olaparib for the respective cancers and later switches to concurrent therapy. This case report discusses a novel combination of targeted drug therapies to treat two aggressive primary cancers simultaneously.

## 2. Case Presentation

### 2.1. Non-Small Cell Lung Cancer (NSCLC)

#### 2.1.1. Initial Presentation and Diagnosis

**Figure 1:**
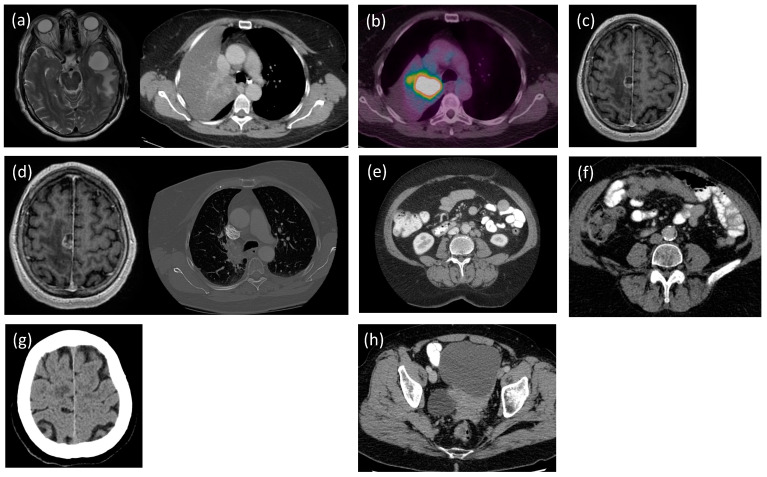
Patient scans. (**a**) The magnetic resonance imaging (MRI) of the head shows cystic metastasis (3.03 × 2.96 × 3.13 cm), and the computed tomography (CT) of the thorax shows the collapsed right upper lobe of the lung (8 March 2017). (**b**) Positron emission tomography (PET) of the whole body demonstrates FDG uptake in level 2 lymph nodes, palatine tonsils, and the tumor in the right upper lobe of the lung (27 April 2017). (**c**) MRI of the head shows the new solid and cystic enhancing mass (3 November 2017). (**d**) CT of the head shows tumor progression (1.6 × 1.5 × 1.9 cm), and CT of the thorax shows central tumor progression (9 March 2018). (**e**) CTs of the abdomen and pelvis show a new liver lesion and increased pelvic lymphadenopathy (10 July 2019). (**f**) CT of the abdomen shows new peritoneal deposits (10 × 3.4 cm) (24 March 2022). (**g**) CT of the head suggests brain lesion progression (8 December 2022). (**h**) CT of the abdomen shows worsening of the ovarian lesion (8.5 × 9.8 cm) and the metastatic deposits in the liver (5 mm) (29 March 2023).

A 75-year-old female with a heavy smoking history (1.5 packs per day, 60 packs per year history) sought consultation for headache and nausea in March 2017. Her computed tomography (CT) scan of the head showed a cystic metastatic lesion, and her CT of the thorax showed a collapse in the right upper lobe of the lung (Figure 1a). She underwent a left temporal craniotomy three days later, and surgical pathology returned as metastatic NSCLC, EGFR-positive. A positron emission tomography (PET) scan revealed a FDG-positive neck lymph node and palatine tonsils (Figure 1b), classifying the patient’s NSCLC at T3N0M1b (stage IV).

#### 2.1.2. Therapeutic Interventions

The patient was started on gefitinib, which worked well for five months. However, this drug was discontinued due to side effects, including elevated liver enzymes and hemorrhagic cystitis. She proceeded with radiation therapy to the chest at 45 Gy in 15 fractions between September and October 2017. Two weeks later, she presented to the emergency room with CNS symptoms, and a CT of the head confirmed a new lesion in the right posterior frontal lobe (Figure 1c). Since the neurosurgery team deemed that this case was inoperable, she received stereotactic radiosurgery (SRS) (18 Gy) in a single fraction. In early 2018, a CT of the head and a CT of the thorax suggested progression of the primary tumor, as well as sites of metastatic disease (Figure 1d). The patient was started on afatinib in March 2018, which stabilized disease progression. In 2020, she developed hypersensitivity to afatinib after 29 months of treatment, which prompted the termination of this drug. She was subsequently started on osimertinib, which was approved due to the hypersensitive reaction to afatinib. The patient reported minimal side effects from osimertinib, aside from occasional nausea that was well controlled with antiemetics, and her quality of life was well maintained.

### 2.2. Ovarian Serous Carcinoma

#### 2.2.1. Initial Presentation and Diagnosis

While her NSCLC was relatively stable throughout 2018, routine CTs of the abdomen and pelvis showed increased paraaortic and pelvic lymphadenopathy in July 2019 (Figure 1e). This progression was suspicious for the development of afatinib resistance in the lung cancer. To determine the T790M mutation status and the eligibility for initiating osimertinib, circulating tumor DNA was performed. The blood test came back negative, yet due to the uncertainty, the patient also underwent a biopsy of her retroperitoneal lymph node. Though negative for T790M lung cancer, the biopsy of her retroperitoneal lymph node returned as metastatic high-grade serous carcinoma. Subsequent molecular analysis of her biopsy specimen confirmed a BRCA2 somatic mutation. Hereditary testing was performed, confirming a germline BRCA2 mutation.

#### 2.2.2. Therapeutic Interventions

The patient was started on osimertinib for her lung cancer shortly before chemotherapy for her ovarian cancer in August 2020. She felt generally unwell with nausea, dehydration, sweating, and irregular bowel movements. Due to the negative side effects, osimertinib was held throughout chemotherapy. The patient received six cycles of carboplatin and paclitaxel from August 2020 to January 2021, with a significant partial response. In light of her concurrent stage IV NSCLC diagnosis, she was not deemed a candidate for surgical cytoreduction. After completion of chemotherapy, consideration was given to olaparib maintenance therapy; however, the decision was made to prioritize the treatment of her stage IV NSCLC with the use of osimertinib. Concurrent use of olaparib and osimertinib has not been described in literature, and all team members had concerns for tolerability.

Upon routine follow-up surveillance imaging in March 2022, new peritoneal deposits were found that suggested progression of the ovarian cancer (Figure 1f). To address the worsening ovarian carcinoma, she underwent another four cycles of carboplatin and liposomal doxorubicin from May to September 2022. After four cycles of chemotherapy, the patient switched to maintenance olaparib. However, there remained a need for the simultaneous management of two primary malignancies while preventing the accumulation of adverse side effects. Therefore, the patient was switching between olaparib and osimertinib every month to treat the ovarian cancer and lung cancer, respectively. Due to the worsening of the brain metastasis from the primary lung cancer at the end of 2022 (Figure 1g), olaparib was held to prioritize the treatment with osimertinib. However, the patient’s rising CA-125 remained a concern, and a CT of the abdomen revealed worsening of the ovarian lesion and metastatic deposits in the liver in March 2023 (Figure 1h). Therefore, the oncology team decided to begin co-prescribing osimertinib and olaparib on 28 June 2023. On 21 September 2023, the olaparib was discontinued, due to potential reduced efficacy and adverse side effects. These included constant nausea, dysgeusia, fatigue, and lack of appetite, which significantly reduced her quality of life.

### 2.3. Follow-Up and Outcomes

Currently, the patient is only on osimertinib. She is followed-up by her oncologists monthly to monitor for side effects and disease progression.

**Figure 2:**
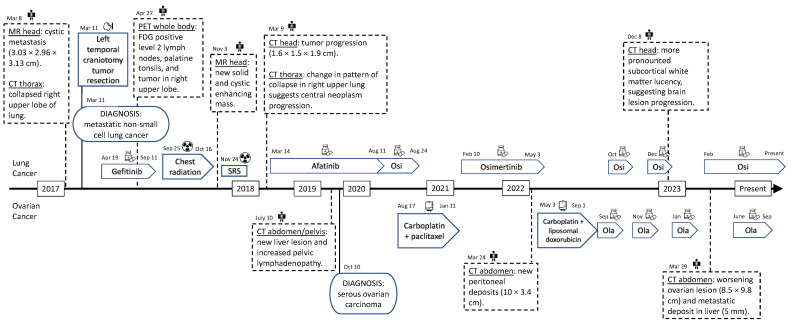
Timeline of the diagnoses and treatments. Osi—osimertinib. Ola—olaparib. SRS—stereotactic radiosurgery. MR—magnetic resonance. CT—computed tomography. PET—positron emission tomography.

Figure 2 shows a full view of the patient’s clinical course. For the summary of treatments for the patient’s lung cancer and ovarian cancer, refer to Table 1.

## 3. Discussion

This case report describes the rare double diagnosis of NSCLC and ovarian serous carcinoma. The patient survived from 2017 to 2023 with two driver mutation-associated cancers. She received radiation therapy for the lung cancer and brain metastasis, chemotherapy for the ovarian cancer, and targeted drug therapies for both malignancies. Given the lung cancer comorbidity, surgical cytoreduction was not recommended for her ovarian cancer. Response rates for cytotoxic chemotherapy are lower, and toxicity profiles are usually worse, compared to targeted therapies in both lung and ovarian cancers. The patient did receive carboplatin and paclitaxel at one point, which would cover both diseases, but the chemotherapy has lower brain penetration than anti-EGFR treatment, which was important in this case. Therefore, targeted therapy was preferred in the longer term. Through the alternation of targeted therapies when one cancer progressed, this led to a significant overall survival (OS) length with tolerable side effects. The use of osimertinib and olaparib led to the extended duration of disease control (DDC). The DDC is the sum of all the progression-free survivals (PFSs) of each interval where the patient has been on the treatment. In the report, the patient’s DDC will be compared with PFS values in literature.

### 3.1. Comparison to Literature

From our case study, the patient experienced a DDC of 37 months on osimertinib (PFS1: 27 months from August 2020 to December 2022; PFS2: 10 months from February 2023 to December 2023), as well as an OS of 40 months. The TREM study showed that the PFS and OS of patients with T790M-positive NSCLC on osimertinib are 10.8 months and 22.5 months, respectively [11]. Compared to combined chemotherapy (pemetrexed plus one of carboplatin or cisplatin), which resulted in a PFS of 4.4 months, patients had significantly better outcomes on osimertinib (*p* < 0.001) [5]. Our patient had an incredible response to osimertinib, as her DDC and OS were nearly doubled, compared to literature. Patients with NSCLC commonly develop CNS metastases, with up to a 65% lifetime incidence reported [5]. The FLAURA trial demonstrated that the frequency of CNS progression was low in the osimertinib group at 6% [7]. Though our patient had CNS progression in December 2022, her treatment with osimertinib was complicated by the need to switch to olaparib every month. In terms of her ovarian cancer, the patient obtained a DDC of 13 months on olaparib (PFS1: 7 months from September 2022 to March 2023; PFS2: 6 months from June 2023 to December 2023). The SOLO-2 study of olaparib maintenance versus a placebo in BRCA-mutated ovarian cancer demonstrated an improvement in PFS from 5.5 months to 19.1 months (hazard ratio 0.30, *p* < 0.0001). Our patient had a shorter time of disease control with olaparib; however, her time on treatment was limited by the need to concurrently treat her stage IV NSCLC.

### 3.2. Alternating and Concurrent Administrations

The initial plan of alternating the osimertinib and olaparib every month aimed to avoid the high risk of side effects, as per the patient’s wish. Since the initiation of her targeted drug therapies, the patient has fortunately experienced minimal toxicities from osimertinib. The most common side effects of osimertinib include diarrhea, rash, nausea, and poor appetite [12]. In August 2020, osimertinib was held for two weeks after initiation, as the patient complained of nausea, dehydration, and fatigue. However, these symptoms were confounded by the chemotherapy for her ovarian cancer, which was started a week after starting osimertinib. When she resumed osimertinib post-chemotherapy, she did not experience those symptoms again, suggesting a good tolerability for this drug. The most common toxicities for olaparib are nausea/vomiting, anemia, and fatigue [13]. Since starting olaparib, the patient complained of nausea/vomiting, poor appetite, dysgeusia, and fatigue. These symptoms continued during the olaparib months but improved during the osimertinib months. With alternating therapy, she had some time in between the olaparib months to experience symptom-free periods. The feasibility of maintaining disease control, for some time, with reasonable toxicity, of two cancers with an alternating schedule that effectively reduced the dose intensity of either drug raises possibilities for the efficient use of targeted drugs in resource-constrained contexts. However, there remained the risk of the progression of one cancer while on the other therapy, which incentivized a more aggressive treatment to target both cancers simultaneously. Understanding the risk of higher toxicity but the importance of controlling both malignancies, the patient agreed to concurrent therapy. While taking the therapies simultaneously, she reported experiencing worse nausea, poor appetite, and fatigue, which also translated to poor functioning and quality of life. From the patient’s perspective, she attributed the symptoms to olaparib, whereas osimertinib did not contribute. In September 2023, the patient’s increasing CA-125 questioned the effectiveness of olaparib. Through shared decision-making, olaparib was discontinued. Fortunately, the adverse side effects subsided. Though the patient expressed fear of the ovarian cancer worsening without therapy, she was able to regain most functions and maintain better quality of life, compared to when she was on olaparib.

### 3.3. Other Drug Combinations

Combining drug therapies may result in favorable outcomes but should be prescribed with caution to monitor for adverse effects and drug-drug interactions. There are no in vitro or in vivo data available on osimertinib and olaparib interactions. In addition, there are no current reports on osimertinib and olaparib combination therapy, yet other drug combinations have been explored. A case report by Zhang et al. detailed the combination of olaparib with dacomitinib (second-generation EGFR-TKI) for the treatment of NSCLC [14]. Interestingly, this patient developed osimertinib resistance but obtained 6 months of PFS and 13 months of OS with olaparib and dacomitinib. Zhang et al. suggested that TKI-resistant NSCLC may be more sensitive to PARP inhibitors, which highlights the potential contribution of olaparib in lung cancer treatment.

## 4. Conclusions

A double diagnosis of primary lung and ovarian cancers is rare. To our knowledge, this is the first report to chronicle the journey of a patient diagnosed synchronously with EGFR-mutated NSCLC and BRCA-mutated ovarian cancer. Research on the combination of therapies specific to lung and ovarian cancers is limited. This case report demonstrated the potential of osimertinib and olaparib combination therapy to treat simultaneous NSCLC and ovarian serous carcinoma, which can reduce risks of progression and reduce patient anxiety. These two drugs could be administered alternatively or concurrently, though the side effect profile was worse for the latter method, which resulted in the termination of the combination therapy. These side effects may significantly impact functioning, which can undermine the patients’ goals if not explored. When presented with complex diagnoses, such as dual malignancies, innovative strategies such as the alternation of therapies and combination trials may be reasonable approaches. However, it is essential to tailor the regimen to the individual and consider patient quality of life when making clinical decisions.

## Figures and Tables

**Table 1:** Summary of the targeted drug therapies, chemotherapies, and radiation therapies. Side effects are reported by the patient.

	Name	Target	Start Date	End Date	Cycles	Dose	Side Effects
Targeted Drug Therapy	Gefitinib	Lung cancer	19 April 2017	11 September 2017	4	250 mg/day, daily	Rash, diarrhea, dysuria, hematuria
	Afatinib	Lung cancer	14 March 2018	11 August 2020	32	20–30 mg/day, daily	Rash, diarrhea, anaphylaxis
	Osimertinib	Lung cancer	10 August 2020	Present	31	80 mg/day, daily	None
	Olaparib	Ovarian cancer	1 September 2022	21 September 2023	6	2 × 150 mg BID, total 300 mg/day	Nausea, vomiting, taste distortion, poor appetite, fatigue
Chemotherapy	Carboplatin + Paclitaxel	Ovarian cancer	17 August 2020	11 January 2021	6	Carboplatin: 5 AUC Paclitaxel: 175 mg/m^2^	Fatigue, nausea, diaphoresis, constipation, diarrhea, dysgeusia, neuropathy
	Carboplatin + Liposomal doxorubicin	Ovarian cancer	3 May 2022	1 September 2022	4	Carboplatin: 5 AUC Liposomal doxorubicin: 30 mg/m^2^	Fatigue, nausea, vomiting, heartburn, diarrhea, dysgeusia, neuropathy
Radiation Therapy	Chest radiation therapy	Lung cancer	25 September 2017	16 October 2017	-	45 Gy in 15 fractions	None
	Stereotactic radiosurgery	Brain metastasis from primary lung cancer	24 November 2017	24 November 2017	-	18 Gy in 1 fraction	Weakness

## Data Availability

Due to the nature of the research, supporting data are not available.

## References

[1] Vogt A., Schmid S., Heinimann K., Frick H., Herrmann C., Cerny T., Omlin A. (2017). Multiple primary tumours: Challenges and approaches, a review. ESMO Open.

[2] Mariniello A., Ghisoni E., Righi L., Catino A., Chiari R., Del Conte A., Barbieri F., Cecere F., Gelibter A., Giajlevra M. (2019). Women with Synchronous or Metachronous Lung and Ovarian Cancer: A Multi-Institutional Report. In Vivo.

[3] Ganti A.K., Klein A.B., Cotarla I., Seal B., Chou E. (2021). Update of Incidence, Prevalence, Survival, and Initial Treatment in Patients With Non–Small Cell Lung Cancer in the US. JAMA Oncol..

[4] Karachaliou N., Fernandez-Bruno M., Bracht J.W.P., Rosell R. (2019). EGFR first- and second-generation TKIs—There is still place for them in EGFR -mutant NSCLC patients. Transl. Cancer Res..

[5] Lee C.-S., Milone M., Seetharamu N. (2021). Osimertinib in EGFR-Mutated Lung Cancer: A Review of the Existing and Emerging Clinical Data. OncoTargets Ther..

[6] Niwa H., Nakahara Y., Sasaki J., Masuda N. (2018). A promising response to osimertinib in a patient with erlotinib-resistant lung adenocarcinoma with an uncommon EGFR mutation. Case Rep..

[7] Soria J.-C., Ohe Y., Vansteenkiste J., Reungwetwattana T., Chewaskulyong B., Lee K.H., Dechaphunkul A., Imamura F., Nogami N., Kurata T. (2018). Osimertinib in Untreated EGFR-Mutated Advanced Non–Small-Cell Lung Cancer. N. Engl. J. Med..

[8] Torre L.A., Trabert B., DeSantis C.E., Miller K.D., Samimi G., Runowicz C.D., Gaudet M.M., Jemal A., Siegel R.L. (2018). Ovarian Cancer Statistics, 2018. CA Cancer J. Clin..

[9] Moore K., Colombo N., Scambia G., Kim B.-G., Oaknin A., Friedlander M., Lisyanskaya A., Floquet A., Leary A., Sonke G.S. (2018). Maintenance Olaparib in Patients with Newly Diagnosed Advanced Ovarian Cancer. N. Engl. J. Med..

[10] Poveda A., Floquet A., Ledermann J.A., Asher R., Penson R.T., Oza A.M., Korach J., Huzarski T., Pignata S., Frielander M. (2021). Olaparib tablets as maintenance therapy in patients with platinum-sensitive relapsed ovarian cancer and a BRCA1/2 mutation (SOLO2/ENGOT-Ov21): A final analysis of a double-blind, randomised, placebo-controlled, phase 3 trial. Lancet Oncol..

[11] Eide I.J.Z., Helland A., Ekman S., Mellemgaard A., Hansen K.H., Cicenas S., Koivunen J., Grønberg B.H., Brustugen O.T. (2020). Osimertinib in T790M-positive and -negative patients with EGFR-mutated advanced non-small cell lung cancer (the TREM-study). Lung Cancer.

[12] Rodier T., Puszkiel A., Cardoso E., Balakirouchenane D., Narjoz C., Arrondeau J., Fallet V., Khoudour N., Guidi M., Vidal M. (2022). Exposure–Response Analysis of Osimertinib in Patients with Advanced Non-Small-Cell Lung Cancer. Pharmaceutics.

[13] Velev M., Puszkiel A., Blanchet B., de Percin S., Delanoy N., Medioni J., Gervais C., Balakirouchenane D., Khoudour N., Pautier P. (2021). Association between Olaparib Exposure and Early Toxicity in BRCA-Mutated Ovarian Cancer Patients: Results from a Retrospective Multicenter Study. Pharmaceuticals.

[14] Zhang H., Wang Y., Wu H., Zhou S., Li S., Meng X., Tao R., Yu J. (2023). Olaparib Combined with Dacomitinib in Osimer-Tinib-Resistant Brain and Leptomeningeal Metastases from Non-Small Cell Lung Cancer: A Case Report and Systematic Review. Front. Oncol..

